# A Rare Cause of Pain in the Oral Cavity: Osteomyelitis of Tori

**DOI:** 10.7759/cureus.66006

**Published:** 2024-08-02

**Authors:** Christopher Aduwari, Amogh Chandupatla, Ramin Javan

**Affiliations:** 1 Department of Radiology, George Washington University School of Medicine and Health Sciences, Washington, DC, USA

**Keywords:** oral tori, torectomy, oral diseases, osteonecrosis, dental caries, exostoses, mandibular tori, osteomyelitis

## Abstract

A 63-year-old man presented with a 1-month history of worsening mouth pain, particularly under the tongue bilaterally, with left more than right. A physical examination revealed multiple dental caries and bilateral mandibular tori, with the left mandibular torus being exquisitely tender to palpation. Lab tests showed increased inflammatory markers in the absence of leukocytosis. A maxillofacial computed tomography scan revealed findings concerning for chronic osteomyelitis with osteolysis of the left mandibular torus. The patient was started on intravenous antibiotics and transferred to another institution for further management through their oral and maxillofacial surgery service. The surgical pathology after torectomy confirmed the diagnosis of acute osteomyelitis with osteonecrosis. Although rare, this case underscores the importance of familiarity with osteomyelitis in tori of the oral cavity, also highlighting the imaging and clinical correlation. Further research is necessary to understand the risk factors and optimal management strategies for similar cases.

## Introduction

Osteomyelitis is defined as an inflammatory process of bone and bone marrow caused by an infectious organism(s) which results in local bone destruction [1]. Its manifestations are varied, depending on the age of the patient, anatomic area of involvement, and specific causative organism [2]. These organisms can be spread contiguously (commonly found in adults) or hematogenously (more commonly found in children) [3]. The infectious process can either be acute or chronic, with acuity dependent on histologic or radiologic findings, rather than the duration of the infection. Acute osteomyelitis is often associated with inflammatory bone changes, with symptoms typically presenting within two weeks of the infection [4]. Chronic osteomyelitis is typically associated with histologic findings of necrotic bone and plain radiograph findings of Inhomogeneous osteosclerosis and/or sequestrum formation, where symptoms may not occur until six weeks after the onset of the infection [5].

Osteolysis or osteonecrosis, on the other hand, refers to a pathologic process that involves the progressive destruction or dissolution of bone. It is a term frequently used in the field of orthopedics and is classically defined in orthopedic literature as peri-prosthetic bone erosion seen as progressive radiolucent lines at the interface of normal bone tissue and the prosthesis [6]. This peri-prosthetic obliteration results from chronic osteoclastic overstimulation at the bone-prosthesis interface [7]. However, osteolysis also occurs in various other situations like osteomyelitis, tumor metastasis (most common in multiple myeloma), and chronic inflammatory conditions such as rheumatoid arthritis (RA). This is because the immune cells and their cytokines influence the activities of osteoclasts, osteoblasts, and osteocytes, with the osteoclast being the main effector cell in focal osteolysis [8]. Of note, anti-resorptive medications, such as bisphosphonates, are known to specifically cause mandibular osteonecrosis, an entity known as medication-related osteonecrosis of the jaw (MRONJ) [9].

Torus mandibularis is a benign, generally asymptomatic, bony sublingual exostosis, typically near the canine and premolar teeth [10]. In addition to torus palatinus and torus maxillaris, torus mandibularis is one of the most common intraoral osseous growths; however, the prevalence varies across different populations with significant heterogeneity in the contributions from race or gender [11]. In most people, tori are asymptomatic, therefore, torectomy is not indicated. However, some people with larger mandibular tori may have obstructive pathology, which can cause significant problems with eating, swallowing, and very commonly, teeth cleaning [12]. In the setting of inappropriate oral hygiene, it is not uncommon to find dental caries, complications for which include apical periodontitis, periostitis, and osteomyelitis. We, therefore, describe the case of a patient who presented with radiologic findings suggestive of left-sided acute to subacute osteomyelitis of a massive mandibular torus in the setting of chronic dental carries, with focal osteonecrosis/osteolysis.

## Case presentation

A 63-year-old male with a past medical history of lung cancer, status post-lobar resection only, presented to our emergency department with mouth pain. The patient stated that his pain felt like a tooth was growing under his tongue, with no mention of any similar episodes in the past. It had started about a month prior and had worsened over the previous two weeks in the absence of any febrile illness. He went to a dentist one day before arriving at our ER and was prescribed penicillin. He denied ever having felt this type of pain before and was unable to eat, with complaints of a choking sensation when trying to swallow saliva. The pain he felt was worse in his left jaw. He denied any chest pain, throat swelling, breathing difficulty, or recent illness. His social history was pertinent for chronic alcohol use and cigarette smoking. At the time of admission, he was vitally stable with a pulse of 86 beats per min and a temperature of 36° Celsius. His physical disposition showed moderate distress. The oropharyngeal exam showed moist mucosa with multiple dental caries bilaterally. The patient's previous dental history prior to this acute episode was unknown. There were two 5 cm mucosal-colored hard masses without mucosal ulceration on the floor of his mouth bilaterally. The mass on the left was severely tender but non-erythematous and non-fluctuant. The rest of the physical exam was within normal limits. His labs showed elevated erythrocyte sedimentation rate (ESR) at 48 mm/hr and a C-reactive protein (CRP) at 15.3 mg/L, in the absence of leukocytosis (WBC at 7.61x10e3/mcL). The rest of his labs were unremarkable.

**Figure 1:**
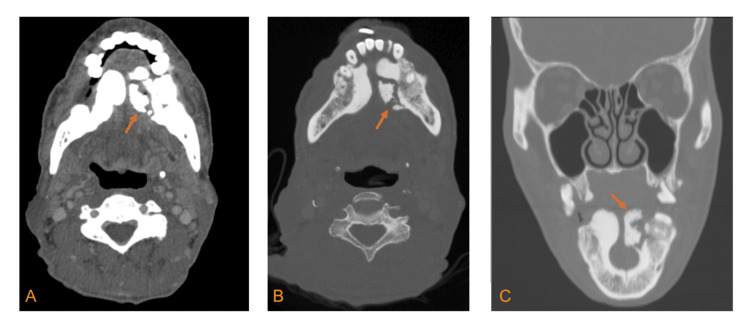
CT images showing bilateral mandibular tori with the left torus showing osteolytic changes A) axial soft tissue window view B) axial bone window view C) coronal bone window view In Figures 1A-1C, the arrows highlight the torus mandibularis with the region of osteonecrosis/osteolysis. No direct communication with periodontal/periapical lucency or a radicular cyst related to any of the adjacent teeth was identified. Mild surrounding fat stranding was present without overt swelling, fluid collection, or disruption of the fat planes. Mild reactive lymphadenopathy in the left submandibular region was noted.

To better characterize the sublingual masses, a maxillofacial computed tomography (CT) scan with contrast was performed and compared to a PET-CT of the skull from five months earlier. The findings of the maxillofacial CT (Figures 1A-1C) included relatively preserved fat planes in the submandibular and submental spaces, indicating a lack of significant inflammatory changes. There was no evidence of a fluid collection, however, there was significant cortical thickening and bony overgrowth of the bilateral mandibular body along the lingual side, compatible with torus mandibularis. The left-sided cortical thickening showed multifocal erosion and a few foci of air within the adjacent soft tissue, which were new findings compared to the positron emission tomography (PET)-CT from four months earlier. No adjacent periodontal disease or evidence of trauma at the site was noted. Dental caries were present elsewhere, however. The patient was started on IV vancomycin and ceftriaxone for empiric coverage of Methicillin-resistant Staphylococcus aureus (MRSA) and Streptococcus mutans, respectively. After assessment by the ENT service, the patient was transferred to another hospital for management by their oral and maxillofacial surgery service. At that hospital, the left mandibular torus was excised, in addition to the removal of two teeth and a biopsy of the floor of the mouth. The patient’s symptoms improved post-torectomy. Surgical pathology of the excised lesion showed irregular osseous fragments measuring 1.7 x 1.9 cm. The final diagnosis was acute osteomyelitis with focal bone necrosis. He was discharged on a course of amoxicillin/clavulanic acid 875-125 mg for 7 days with a recommendation for follow-up with oral surgery.

## Discussion

The incidence of chronic osteomyelitis has steadily increased due to the rising prevalence of risk factors such as diabetes, peripheral vascular disease, focal trauma, and IV drug use. The availability of advanced imaging techniques like magnetic resonance imaging and bone scintigraphy has enhanced diagnostic precision and the ability to characterize the infection when correlated with clinical signs and lab evaluation [4]. Our patient presented with mouth pain and tenderness of the left floor of the mouth tenderness on physical examination, and in their lab work, although there was no leukocytosis, ESR and CRP levels were more than double the upper limits of normal, indicating a systemic inflammatory state. Given the patient opted out of receiving chemotherapy or radiation after his tumor was excised, immunosuppression was less likely. The initial differential diagnosis then included infectious etiology, malignancy, and cyst. The maxillofacial CT scan ruled out a cyst but revealed findings concerning for osteomyelitis. The patient had a normal white blood cell count, although this finding is not uncommon in chronic osteomyelitis. Typically, the gold standard diagnostic test to confirm osteomyelitis after positive radiologic findings is a bone biopsy with culture and histology. However, as recommended by our ENT service, the patient was transferred to another institution for surgical evaluation by oral and maxillofacial surgery, with the rationale being that excision of the lesion with an intra-procedural biopsy was the next likely outcome. Given the high index of suspicion for an infectious etiology, the team aimed to minimize the risk of concomitant osteonecrotic spread to the adjacent jaw and soft tissue spaces and administered intravenous antibiotics. Prior reports had shown that pre-biopsy antibiotics did not affect the proportion of positive cultures or change the bacterial yield of the biopsy specimen [13]. Pending imminent transfer, the patient was started on empiric therapy with MRSA and gram-negative flora coverage, given that the working diagnosis was chronic osteomyelitis.

Chronic osteomyelitis is a disease that is extremely difficult to treat because there is an increased risk of relapsing infection, so patients usually require a combined medical and surgical approach [14]. Radiographs are especially important in procedural planning because chronic advanced osteomyelitis typically shows significant osteolytic changes of bone, which in turn dictates the level of intervention. The extent of osteolysis is a non-negligible radiographic data point that helps surgeons in planning for bone debridement [8]. Our patient’s CT showed chronic multifocal erosions with what seemed to be a geographic pattern of bony destruction of the left mandibular torus, highly suggestive of infection-related osteolysis. The underlying etiology likely is bacterial translocation secondary to chronic dental caries. In prior research, odontogenic infections have been found to be capable of spreading to nearby osseous structures like the mandible and maxilla, and mandibular tori are no exception. It is because of the rarity of these tori in the general population that we see a reduced incidence of damage to them, let alone instances of osteolysis. Hence, osteomyelitis and osteolysis of mandibular tori are not well documented in the literature. Instead, the literature mostly views these tori as incidental findings on imaging, and nearly all patients who have them are asymptomatic [12].

One consideration is that prior reports have shown that mechanical trauma to these tori can cause mucosal damage and subsequent erosive changes [15]. In our patient, even though he noted that there was no inciting traumatic event, there is no definitive proof that the contributions of trauma-related osteolysis can be considered minor. Repeated mechanical stress from daily mastication may have contributed to his presentation. We also cannot exclude other risk factors such as chronic alcohol use and smoking. Chronic alcohol use can impair immune function and wound healing, making the oral cavity more susceptible to infection and subsequent osteolysis. In the setting of these risk factors and the patient’s clinical presentation, the most likely diagnosis was osteomyelitis with osteolytic changes, which was confirmed in the post-surgical pathology report. With an improvement in the patient’s symptoms following torectomy, the care provided can be considered appropriate, and this management approach can serve as a reference for similar cases in the future.

## Conclusions

The clinical significance of this case lies in its rarity and its contribution to existing literature, offering insights that may enhance preventative practices. For primary prevention, a multidisciplinary approach involving dental specialists, radiologists, and infectious disease experts is recommended to ensure the prompt treatment of odontogenic infections in patients with maxillary and mandibular tori. At this time, we cannot characterize the absolute risk of developing an adjacent infection in any bony exostoses in the setting of dental caries, but we know it is possible in this population. To answer this question, more research on this rare manifestation is needed. Regarding tertiary prevention, we believe that no significant changes are warranted. The working diagnosis in our patient was osteomyelitis, but given the broad differential diagnosis (with malignancy being a critical consideration), biopsy and surgical intervention will likely remain necessary.
